# Effect of adhesive remnant removal on enamel topography after bracket debonding

**DOI:** 10.1590/2176-9451.19.6.105-112.oar

**Published:** 2014

**Authors:** Larissa Adrian Meira Cardoso, Heloísa Cristina Valdrighi, Mario Vedovello, Américo Bortolazzo Correr

**Affiliations:** 1 UNIARARAS, Hermínio Ometto Foundation, DDS, Hermínio Ometto Foundation, UNIARARAS; 2 UNIARARAS, Hermínio Ometto Foundation, Graduate Dental Program, Professor, Graduate Dental Program, Hermínio Ometto Foundation, UNIARARAS; 3 São Leopoldo Mandic, Masters Program in Dentistry, Professor, Masters Program in Dentistry, São Leopoldo Mandic; 4 UNIARARAS, Hermínio Ometto Foundation, Masters Program in Dentistry and at the Department of Dental Material, Professor, Masters Program in Dentistry and at the Department of Dental Material, Hermínio Ometto Foundation, UNIARARAS

**Keywords:** Orthodontic brackets, Dental enamel, Device removal

## Abstract

**INTRODUCTION:**

At orthodontic treatment completion, knowledge about the effects of adhesive remnant removal on enamel is paramount.

**OBJECTIVE:**

This study aimed at assessing the effect of different adhesive remnant removal methods on enamel topography (ESI) and surface roughness (Ra) after bracket debonding and polishing.

**METHODS:**

A total of 50 human premolars were selected and divided into five groups according to the method used for adhesive remnant removal: high speed tungsten carbide bur (TCB), Sof-Lex discs (SL), adhesive removing plier (PL), ultrasound (US) and Fiberglass burs (FB). Metal brackets were bonded with Transbond XT, stored at 37^o^C for 24 hours before debonding with adhesive removing plier. Subsequently, removal methods were carried out followed by polishing with pumice paste. Qualitative and quantitative analyses were conducted with pre-bonding, post-debonding and post-polishing analyses. Results were submitted to statistical analysis with F test (ANOVA) and Tukey's (Ra) as well as with Kruskal-Wallis and Bonferroni tests (ESI) (P < 0.05).

**RESULTS:**

US Ra and ESI were significantly greater than TCB, SL, PL and FB. Polishing minimized Ra and ESI in the SL and FB groups.

**CONCLUSION:**

Adhesive remnant removal with SL and FB associated with polishing are recommended due to causing little damage to the enamel.

## Abstract (pt)

**INTRODUÇÃO:**

na finalização do tratamento ortodôntico, torna-se relevante o conhecimento da ação dos métodos de remoção do remanescente resinoso sobre o esmalte.

**OBJETIVO:**

o objetivo do estudo foi avaliar o efeito de métodos de remoção do remanescente de resina após a descolagem do braquete e do polimento na rugosidade (Ra) e topografia (ESI) do esmalte.

**MÉTODOS:**

foram selecionados 50 pré-molares humanos, divididos em cinco grupos, de acordo com o método empregado para a remoção da resina residual: broca carbide tungstênio em alta rotação (BCT), discos Sof-Lex (SL), alicate removedor de resina (AL), ultrassom (US) e pontas Fiberglass (PF). Braquetes metálicos foram colados com Transbond XT, armazenados a 37° por 24 horas antes da descolagem com alicate removedor de braquete, sendo aplicados posteriormente os meios de remoção e executado o polimento com pasta de pedra-pomes. Realizou-se análises qualiquantitativas, com avaliações antes da colagem dos braquetes, após a descolagem e após o polimento, sendo os valores obtidos submetidos à análise estatística com teste F (ANOVA), de Tukey (Ra) e testes de Kruskal-Wallis e Bonferroni (ESI) (p < 0,05).

**RESULTADOS:**

a Ra e o ESI do US foram significativamente maiores do que BCT, SL, AL e PF. O polimento reduziu a Ra e ESI dos grupos SL e PF.

**CONCLUSÃO:**

a remoção do adesivo resinoso com SL e PF associados ao polimento são os métodos mais indicados por ocasionarem as menores alterações do esmalte.

## INTRODUCTION

Orthodontic accessories were, for many years, featured by band welding systems^1^. In 1955, Buonocore enabled orthodontic therapy to be conducted with restorative material bonded over enamel surface.^2^ Later on, Newman allowed metallic material to be bonded over enamel surface,^1^^,^ 3 thereby offering many benefits provided by direct bonding: improved esthetics and performance, better hygiene, low costs, caries risk reduction, and accurate bracket positioning.^1^^,^ 4 ^,^ 5 ^,^ 6

The bonding process is no longer an issue. The greatest challenges are with regard to accurate removal of adhesive remnant,^3^^,^ 4 ^,^ 7 ^-^ 10 so as to avoid not only irreversible iatrogenic injuries, such as rough surfaces, vertical cracks, pulp necrosis, loss of the external surface rich in fluorine (20 µm), but also the presence of adhesive remnant at the adhesion area.^2^^,^ 4 ^,^ 11 ^,^ 12 ^,^ 13 These injuries can be caused by inappropriate removal techniques, prophylaxis with abrasives, bonding material, acid conditioning and color similarity between residual adhesive and enamel.^11^^,^ 12 ^,^ 14 ^,^ 15

The literature presents a great variety of mechanical removal methods, namely: adhesive removing plier,^1^^,^ 3 ^,^ 5 ^,^ 8 ^,^ 12 ^,^ 16 high and low speed tungsten carbide bur, ,^1^^,^ 3 ^,^ 5 ^,^ 6 ^,^ 8 ^,^ 9 ^,^ 10 ^,^ 14 ^-^ 19 laser application,^15^^,^ 16 Shofu bur,^1^^,^ 2 ^,^ 3 ^,^ 8 ^,^ 12 ^,^ 16 Sof-Lex^(r)^ discs,^5^^,^ 6 ^,^ 15 ^,^ 16 ^,^ 18 ^,^ 20 fiberglass burs^12^^,^ 19 and ultrasound.^5^^,^ 10 ^,^ 14 ^,^ 17 For polishing, rubber cup with pumice and water^5^^,^ 6 ^,^ 7 ^,^ 9 ^,^ 18 as well as diamond paste^5^ are used.

Nevertheless, no consensus has yet been reached in the literature regarding the most efficient and safe technique to this end.^3^ Considering that the aforementioned tools are largely used by orthodontists, scientific knowledge about these techniques as well as their biological cost to tooth structure is essential. As a result, there is a great need for choosing effective removal techniques in order to cause the least damages to the patient at the end of treatment and, whenever possible, preserve the tooth original condition. 1 ^,^ 2 ^,^ 3 ^,^ 6 ^,^ 7 ^,^ 9 ^,^ 11 ^,^ 12 ^,,^ 13 ^,^ 16 ^-^ 19

This study aimed to conduct an *in vitro* assessment so as to investigate the effect of adhesive remnant removal after bracket debonding and polishing on roughness (Ra) and enamel topography (ESI). 

## MATERIAL AND METHODS

A total of 50 premolars obtained from a teeth bank were submitted to local Institutional Review Board. The study was approved under protocol #162.677.

The teeth were standardized with minimal previous lesions, based on exclusion criteria (fracture, caries, restorations or coronal cracks, orthodontic and endodontic treatment). Subsequently, they were washed in tap water and cleaned with periodontal curettes,^2^^,^ 8 submerged in distilled water (ISO 3696:1987)^21^ and stored at -8°C.^3^ The immersion means was weekly changed in order to prevent dehydration and bacterial growth, and to improve adhesive strength.^3^^,^ 21

The sample was divided into five groups (n = 10) according to the adhesive remnant removal method used.

Specimens were manufactured with the roots cut in the cement-enamel junction using a double-sided diamond disc (KG-Sorensen, Rio de Janeiro, RJ, Brazil). The crowns were embedded in PVC and fixed with polystyrene adhesive (Resina Cristal, Piraglass, Piracicaba, SP, Brazil) with the buccal surface exposed. That was where the bracket bonding area was delimited with #0 brush (Marta-Tigrei bristle) and nail polish (color: red - Colorama). This procedure allowed both rugosimetric analysis and digital photographs to be taken in the enamel region.

Roughness (Ra) quantitative analysis was carried out by means of a rugosimeter (Surfcorder, mod. 1700, Japan), with horizontal readings towards the center of the delimited surface (distance = 2.5 mm; speed = 0.1 mm; s-Cut Off = 0.25 mm). Qualitative analysis was conducted with digital photographs (Sony Cyber-shot Digital Camera /12.1 Mp/zoom 1.5 x) under magnification of 40 x and 100 x obtained by stereomicroscopic imaging (Carl Zeiss-Citoval, mod. 2, Germany).

**Figure 1.**
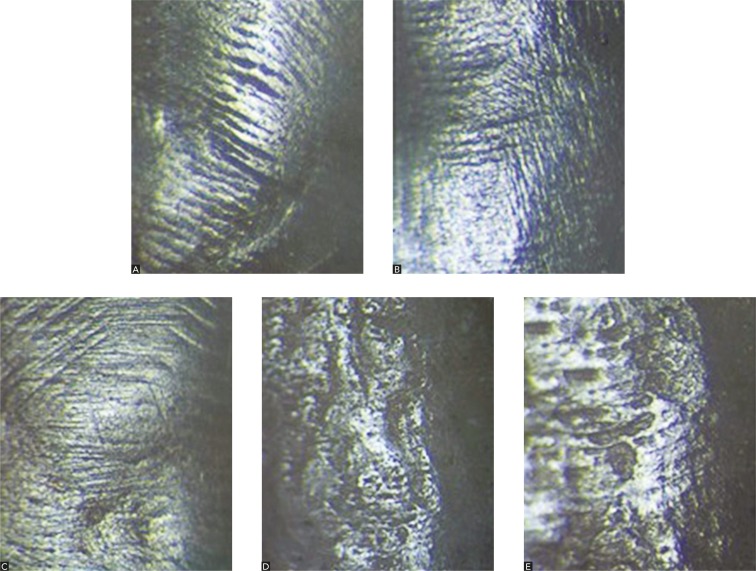
Digital photographs (100 x) for ESI classification: A) Score 0; B) Score 1; C) Score 2; D) Score 3; and E) Score 4.

Data were transferred to a computer (JPG format with 12-MB resolution), and ESI classified according to Zachrisson and Arthun's^17^ criteria (Fig 1):

" Score 0: perfect surface (with no scratches, intact enamel).
" Score 1: regular surface (minor scratches and some healthy enamel).
" Score 2: acceptable surface (many deep scratches, absent healthy enamel).
" Score 3: defective surface (many large, deep scratches, absent healthy enamel).
" Score 4: unacceptable surface (large, deep scratches and deeply marked surface).

Before bracket bonding, prophylaxis was carried out with pumice paste (Extra-fine/SS White, Rio de Janeiro, RJ, Brazil) and rubber cup (Microdont, São Paulo, SP, Brazil) during 10 seconds. Conditioning was carried out with 37% phosphoric acid (FGM, Joinville, SC, Brazil) for 30 seconds, washed for other 30 seconds. Transbond XT Light Cure Adhesive Primer (3M/ Unitek, Monrovia, CA, USA) was applied, dried with compressed air during 10 seconds and light-cured by halogen lamp (Light Unit, Degussa, USA) with irradiance of 500 mW/cm^2^. Subsequently, composite resin Transbond XT Light Cure Adhesive Primer (3M Unitek) was applied to metal brackets base (Edgewise, slot 022-in/Morelli, Sorocaba, SP, Brazil) which was compressed against the enamel surface. This allowed excess removal by light-curing processes with halogen lamp during 10 seconds on each side of the bracket.

The sample was stored in distilled water in the sterilizer (FANEM, São Paulo, SP, Brazil) at 37°C during 24 hours before bracket removal with orthodontic plier (346R/ICE, Cajamar, SP, Brazil), in which case the winglets were perpendicularly pressed against the slot axis.

Enamel surface was analyzed by stereomicroscopic analysis (Carl Zeiss-Citoval, mod. 2, Germany) under 40 x and 100 x magnification. Cases in which adhesive was not adhered to enamel were discarded.

**Figure 2.**
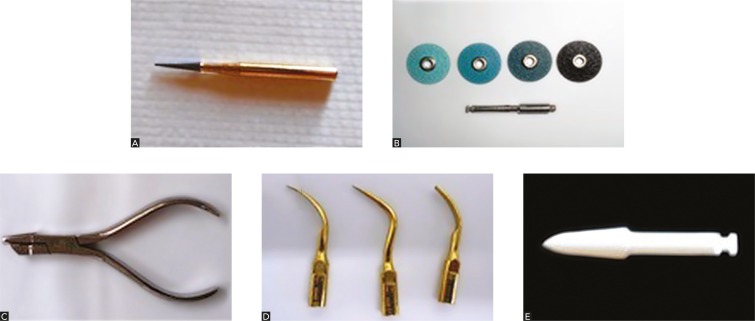
Adhesive remnant removal methods: A) High speed tungsten carbide drill (TCB); B) Sof-Lex discs (SL); C) Adhesive removing plier (PL); D) Ultrasound (US); and E) Fiberglass burs (FB).

Adhesive remnant removal methods were (Fig 2):

" TCB group: High speed tungsten carbide drill with 30 blades (#9214 FF/JET) during 30 seconds.
" SL group: Low speed large, middle, fine and super fine Sof-Lex discs (3M ESPE) for 40 seconds.
" PL group: Adhesive removing plier (#193/ICE) in 10 seconds.
" US group: Ultrasound on large, middle and fine tips (#02, 01 and 10-P/Gnatus) during 90 seconds.
" FB group: Fiberglass burs in low speed with water for 20 seconds (#02/TDV).

After remnant adhesive removal, new assessments of enamel surface roughness (Ra) as well as new photographs (ESI) were obtained. The presence of adhesive remnant was visually inspected under dental reflector light (Dabi Atlante, Ribeirão Preto, SP, Brazil). Polishing was carried out with pumice paste (SS White, Rio de Janeiro, RJ, Brazil) and rubber cup (Microdont, São Paulo,/SP, Brazil) during 10 seconds. After polishing, new assessments of enamel surface roughness (Ra) as well as new photographs (ESI) were obtained. 

Qualitative and quantitative analyses were performed before bracket bonding (initial Ra and ESI), after debonding (adhesive removal Ra and ESI), and after polishing (Ra and ESI polishing). Data were statistically assessed. Roughness (Ra) values were submitted to analysis of variance (ANOVA F-test) and Tukey's test, whereas ESI (scores) values were submitted to Kruskal-Wallis and Bonferroni test. Significance level was set at 5%.

## RESULTS

Analysis of variance showed that enamel's Ra was significantly influenced by the remnant adhesive removal method used (P < 0.001). 

**Figure 3.**
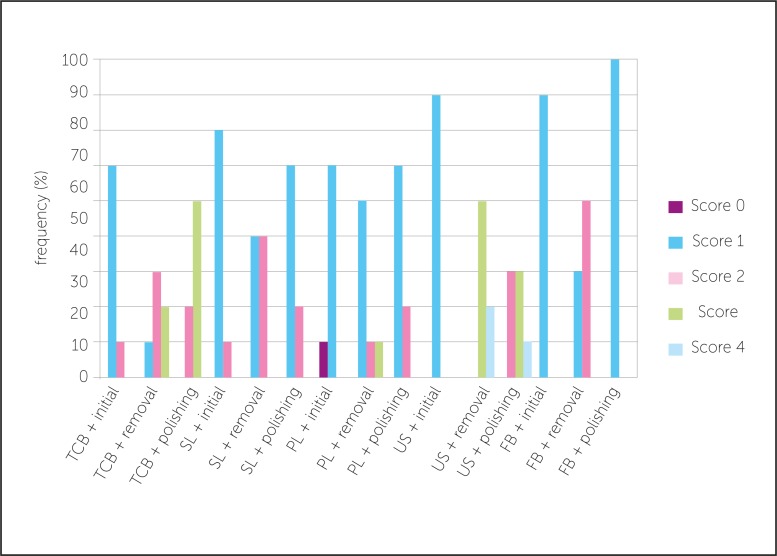
Distribution of ESI frequency. Score 0 = Perfect, 1 = regular; 2 = acceptable; 3 = defective; 4 = unacceptable. TCB: High speed tungsten carbide drill; SL: Sof-Lex discs; PL: Adhesive removing plier; US: Ultrasound; and FB: Fiberglass burs.

Initial Ra and ESI were significantly similar in all groups (Tables 1 and 2). Regular topography prevailed (Score 1) (Fig 3).

**Table 1.** Roughness means and standard deviation for different adhesive removal methods at different assessment times.

Removal methods	Assessment times		
	Initial	Adhesive removal	Polishing
	Mean ± SD	Mean ± SD	Mean ± SD
TCB	2.0309 ± 0.5795^aA^	0.8291 ± 0.2983^bB^	1.0151 ± 0.3226^bB^
SL	1.5500 ± 0.4318^aA^	0.4701 ± 0.0674^bB^	0.4401 ± 0.1977^bB^
PL	1.4118 ± 0.3315^aB^	1.7401 ± 0.0339^aAB^	2.0909 ± 0.7268^aA^
US	1.5200 ± 0.4081^aB^	2.2601 ± 0.5544^aA^	1.9793 ± 0.5369^aAB^
FB	1.7803 ± 0.6298^aA^	0.7456 ± 0.2319^bB^	0.7362 ± 0.1647^bB^

Different lower case letters in columns and capital letters in line are meaningfully different by Tukey's test (P < 0.05). TCB: High speed tungsten carbide drill; SL: Sof-Lex discs; PL: Adhesive removing plier; US: Ultrasound; and FB: Fiberglass burs.

**Table 2.** Kruskal-Wallis analysis.

Groups	Median	25%	75%	Mean	Statistics
TCB + initial	1	1	1	1.1	d
TCB + removal	2	2	3	2.3	abc
TCB + polishing	3	2	3	2.5	ab
SL + initial	1	1	1	1.2	d
SL + removal	1.5	1	2	1.5	bcd
SL + polishing	1	1	2	1.3	cd
PL + initial	1	1	1	0.9	d
PL + removal	1	1	2	1.6	bcd
PL + polishing	1	1	2	1.3	cd
US + initial	1	1	1	1.1	d
US + removal	3	3	4	3.2	a
US + polishing	3	2	3	2.8	a
FB + initial	1	1	1	1.1	d
FB + removal	2	1	2	1.6	bcd
FB + polishing	1	1	1	1	d

TCB: High speed tungsten carbide drill; SL: Sof-Lex discs; PL: Adhesive removing plier; US: Ultrasound; and FB: Fiberglass burs.

US Ra and ESI were greater than the other methods (TCB, SL, PL and FB) with no difference among methods (P < 0.05). Initial Ra was greater than that showed after adhesive removal and after polishing in TCB, SL and FB groups (P < 0.05). Final PL Ra was significantly greater after polishing in comparison to initial Ra. After adhesive removal, Ra was significant greater in comparison to initial Ra (P < 0.05) for the US group. After adhesive removal, TCB and US methods caused more damages to dental enamel; thus, acceptable surfaces prevailed (Score 2).

Polishing was not significant in repairing Ra caused by removal methods (P < 0.05). However, in SL, FB and TCB groups, polishing reduced Ra when compared to initial Ra (P < 0.05), in addition to recovering SL, FB and PL initial quality (ESI), although for TCB and US the polish effect was useless in restoring initial enamel features. Regular surfaces prevailed (Score 1).

## DISCUSSION

Direct bracket bonding on enamel surface contributed to simplify bonding and debonding protocols.^1^^,^ 2 However, after finishing orthodontic treatment, the aim is to restore initial topographic quality,^8^^,^ 18 since irreversible iatrogenic lesions might be caused (5.4%)^6^^,^ 13 due to a number of different factors, as reported in the literature.^11^^,^ 12 Adhesive remnant and damages caused to the enamel structure^3^^,^ 19 are unavoidable regardless of the type of bracket and the removal technique. 

Adhesive remnant removal with rotating instruments causes enamel erosion at both high (19.2 µm) and low (11.3 µm) speed.^18^ Nevertheless, the latter causes more damage to pulp vitality^6^^,^ 8 due to heat produced by the lack of air/water refrigeration. On the contrary, when manual instruments are used, prudence regards to force application is recommended in order to avoid enamel loss.^11^

Ideal removal material hardness has to be greater than the adhesive remnant and lower than the enamel structure. 

The results yielded by the present research were based on roughness and enamel topographic quality parameters and allowed comparison between different methods and potential individual variables; thereby proving unfeasible to determine the best or worst removal method.

Adhesive remnant removal was unfavorable in US groups due to presenting a significant increase in Ra. Conversely, AL groups presented a non-significant increase in Ra when removal methods were used.

US was considered harmful due to presenting defective and unacceptable surfaces with large and deep scratches. These findings are similar to those by Hosein, Sheirriff and Ireland^10^ as well as Ireland, Hosein and Sheirriff,^14^ and are due to difficulty in removing adhesive remnants in consequence of the active insert tip not covering the entire work area. This requires a higher number of applications and more treatment time for complete reduction. Hardness of the tool used is also responsible for the aforementioned results, as it is higher than that of the adhesive remnant and the enamel, thereby causing enamel prism to break with vibrations. 

Although PL produced large, deep vertical stretches and did not completely remove adhesive remnants, the conditions initially observed (regular topography) remained, thereby corroborating Pignatta et al.^7^ This technique had good performance due to being less invasive and providing more comfort to the patient as a result of relatively absence of vibrations when compared to TCB^4^ and US activation. In addition, it produced little enamel roughness and demanded minimal time for reducing adhesive remnant, as stated by Hosein, Sheirriff and Ireland,^10^ Albuquerque et al^8^ and Tavares,^3^ and in disagreement with Miksic, Slaj and Mestrovic^4^ and Rouleau Jr, Marshall Jr and Cooley^9^ who describe this method as the worst choice.

Those visible injuries were caused due to enamel surface convexity while the plier was being supported by the occlusal surface so as to allow the flat active tip to remove residual adhesive by compression.^8^ This procedure also caused enamel thickness wear (µm) possibly detected by the rugosimeter, a manual tool that when subjected to excessive tension can lead to remarkable enamel delamination in comparison to other methods.^11^

The most favorable methods were TCB, SL and FB, as they had Ra reduction (P < 0.05) in comparison to initial Ra. The least Ra was found in the SL method, which is in accordance with Eliades et al.^20^

In comparison to SL and FB, the TCB method showed higher surface variance for marks as well as deep and large scratches (acceptable and defective surfaces). These damages were assigned to higher TCB hardness in comparison to adhesive remnant and enamel, which caused subjacent enamel loss^10^^,^ 14 ^,^ 17 after residual adhesive removal when TCB was operated at high speed. This method, however, causes regular thickness wear (µm), which renders its identification unfeasible by the rugosimeter. Nevertheless, the TCB method had minimal level of Ra,^16^ and proved to be an effective method^8^^,^ 17 ^,^ 18 that causes less damage with faster performance.^1^^,^ 6 Conversely, Karan, Kircelli and Tasdelen^19^ as well as Tavares^3^ assert that this method, when compared to fiberglass burs as well as other methods, presents increased Ra.

Considerable incidence of acceptable surfaces with irregularities (deep scratches and a significant number of marks) was observed in SL and FB. However, SL preserved the initial features of experimental unities in 50% of cases (regular surface). These are considered desirable effects when compared to some researches^5^^,^ 6 ^,^ 15 ^,^ 18 that attribute decrease in surface variations to fine and ultra-fine discs which reduce scratches caused by greater granulation discs (G-M). Nevertheless, it is a complex method for a practical procedure, as its four discs require extensive performance time once it passes through bonding^2^^,^ 15 areas in sequence. 

In accordance with Karan, Kircelli and Tasdelen,^19^ FB is recommended for adhesive remnant removal due to requiring less treatment time as a result of its ability of clearly differentiating adhesive from enamel, quickly wearing it without causing any lesions when in contact with the surface. This is due to the glass fibers which are broken into fragments during abrasive movement and expel the adhesive part by part through grinding. After the procedure, the glass fiber segment is available again, and is improved within the same period. 

On the other hand, Rastelli^12^ asserts that no damage (scratches, cracks or wear) were found on enamel surface. Moreover, Karan, Kircelli and Tasdelen^19^ report that removal by means of this method is delayed more than with TCB. 

Pumice paste polishing is beneficial, fast and pleasant for the patient, as it straightens the majority of rough areas, giving special shine and decreasing abrasive marks.^5^^,^ 6 ^,^ 7 ^,^ 9 ^,^ 18

For SL, FB and TCB, polishing reduced Ra significantly when compared to initial Ra. In addition, it restored SL and FB initial quality (regular surface). It is considered particularly advantageous for eliminating surface changes without injuring pulp tissues and causing minimal enamel loss in conformance with Zarrinnia, Eid, and Kehoe^18^ as well as Campbell.^6^ In the TCB group, deep, large scratches remained due to pumice abrasiveness, thereby making restoration of early features (regular topography) unfeasible and featuring defective surfaces. 

PL group resulted in significant increase of Ra in comparison to natural tooth. Although early enamel quality was restored (regular topography) and large scratches were eliminated, as in agreement with Ryf et al,^11^ deep scratches remained, similarly to Rouleau Jr, Marshall Jr and Cooley^9^ and Pignatta et al.^7^ Enamel layer remained with irregular thickness (µm), which was highlighted by the rugosimeter as Ra increase. 

US group had Ra significantly increased after polishing when compared to initial Ra. Moreover, although it decreased defective and unacceptable surfaces, it did not restore enamel initial features (regular surfaces).

According to some researchers,^11^ no important variances in Ra changes were found after polishing when comparing the interaction among TCB, SL and FB groups and between PL and US groups.

When comparing different removal methods, we found that polishing does not significantly increases or reduces Ra. For this reason, the polishing procedure is optional, as stated by Zachrisson and Arthun.^17^

This fact can be explained by the specific system of the polishing tool with its rough and porous fragments producing low abrasive power. Similarly, differences in TCB blades also affect the dental structure.^11^^,^ 17

Thus, orthodontists should attempt to choose a suitable protocol based on scientific evidence for adhesive remnant removal and initial tooth features restoration so as to avoid undesirable results, reach professional and patient's goals and ensure satisfactory, conservative, successful treatment outcomes.^2^^,^ 5 ^,^ 13

## CONCLUSIONS

Based on the results of this study it is reasonable to conclude that

1. All adhesive remnant removal methods changed enamel topography and roughness.
2. The US method is unsuitable to remove composite resin.
3. The methods of choice, in decreasing order, are: SL, FB, TCB and PL.
4. Pumice paste polishing was insignificant in restoring enamel initial conditions. Therefore, it is optional.
5. SL and FB protocols are recommended in association with polishing due to being capable of restoring enamel initial conditions.
